# A systematic search strategy identifies cubilin as independent prognostic marker for renal cell carcinoma

**DOI:** 10.1186/s12885-016-3030-6

**Published:** 2017-01-04

**Authors:** Gabriela Gremel, Dijana Djureinovic, Marjut Niinivirta, Alexander Laird, Oscar Ljungqvist, Henrik Johannesson, Julia Bergman, Per-Henrik Edqvist, Sanjay Navani, Naila Khan, Tushar Patil, Åsa Sivertsson, Mathias Uhlén, David J. Harrison, Gustav J. Ullenhag, Grant D. Stewart, Fredrik Pontén

**Affiliations:** 1 Department of Immunology, Genetics and Pathology, Science for Life Laboratory, Uppsala University, Uppsala, Sweden; 2 Department of Oncology, Radiology and Radiation Science, Uppsala University, Uppsala, Sweden; 3 MRC Human Genetics Unit, University of Edinburgh, Edinburgh, UK; 4 Edinburgh Urological Cancer Group, Institute of Genetics and Molecular Medicine, University of Edinburgh, Edinburgh, UK; 5 Atlas Antibodies AB, Stockholm, Sweden; 6 Lab Surgpath, Mumbai, India; 7 Science for Life Laboratory, Royal Institute of Technology, Stockholm, Sweden; 8 School of Medicine, University of St. Andrews, St. Andrews, UK; 9 Department of Immunology, Genetics and Pathology, Rudbeck Laboratory, Dag Hammarskjölds Väg 20, SE-751 85 Uppsala, Sweden; 10 Academic Urology Group, University of Cambridge, Box 43, Addenbrooke’s Hospital, Cambridge Biomedical Campus, Hill’s Road, CB2 0QQ Cambridge, UK

**Keywords:** Cubilin, Renal cell carcinoma, Independent prognostic biomarker, Immunohistochemistry

## Abstract

**Background:**

There is an unmet clinical need for better prognostic and diagnostic tools for renal cell carcinoma (RCC).

**Methods:**

Human Protein Atlas data resources, including the transcriptomes and proteomes of normal and malignant human tissues, were searched for RCC-specific proteins and cubilin (CUBN) identified as a candidate. Patient tissue representing various cancer types was constructed into a tissue microarray (*n* = 940) and immunohistochemistry used to investigate the specificity of CUBN expression in RCC as compared to other cancers. Two independent RCC cohorts (*n* = 181; *n* = 114) were analyzed to further establish the sensitivity of CUBN as RCC-specific marker and to explore if the fraction of RCCs lacking CUBN expression could predict differences in patient survival.

**Results:**

CUBN was identified as highly RCC-specific protein with 58% of all primary RCCs staining positive for CUBN using immunohistochemistry. In venous tumor thrombi and metastatic lesions, the frequency of CUBN expression was increasingly lost. Clear cell RCC (ccRCC) patients with CUBN positive tumors had a significantly better prognosis compared to patients with CUBN negative tumors, independent of T-stage, Fuhrman grade and nodal status (HR 0.382, CI 0.203–0.719, *P* = 0.003).

**Conclusions:**

CUBN expression is highly specific to RCC and loss of the protein is significantly and independently associated with poor prognosis. CUBN expression in ccRCC provides a promising positive prognostic indicator for patients with ccRCC. The high specificity of CUBN expression in RCC also suggests a role as a new diagnostic marker in clinical cancer differential diagnostics to confirm or rule out RCC.

**Electronic supplementary material:**

The online version of this article (doi:10.1186/s12885-016-3030-6) contains supplementary material, which is available to authorized users.

## Background

The Human Protein Atlas project has generated a comprehensive map of global gene expression patterns in normal tissues [1]. Through integration of antibody-based, spatial proteomics and quantitative transcriptomics, expression and localization of more than 90% of all human protein-coding genes have been analyzed. Whereas the majority of proteins show a widespread expression profile, subsets of tissue-enriched proteins have been defined [2], including proteins with enriched expression in the kidney [3]. To facilitate screening and discovery efforts for cancer-relevant proteins, the Human Protein Atlas also contains immunohistochemistry-based protein expression profiles for the 20 most common forms of cancer [4].

Renal cell carcinoma (RCC) is the most common type of cancer affecting the kidney. Several histological subtypes of RCC have been defined, the most frequent being clear cell RCC (ccRCC) [5]. Diagnosis and subtyping of RCC are achieved through the morphological analysis of tumor sections. The application of immunohistochemistry (IHC) can reveal important additional clues during the diagnostic work-up. A variety of antibodies have been described to guide pathologists during the diagnosis of distant metastases from the kidney, to distinguish primary RCCs from benign mimics, and to differentiate RCC from malignancies derived from other retroperitoneal structures [6]. Most recently, PAX8 and PAX2 have shown improved RCC-specificity over the traditionally used RCC markers CD10 and RCC monoclonal antibody, although several female genital tract and thyroid tumors stain positive for both markers [7, 8].

The clinical risk stratification of RCC patients relies heavily on the assessment of histopathological parameters. Clear cell histology is significantly associated with a more aggressive disease progression and reduced overall survival [5]. For the prediction of recurrence in patients with localized ccRCC, algorithms were developed by teams at Memorial Sloan-Kettering Cancer Center (based on tumor stage, nuclear grade, tumor size, necrosis, vascular invasion and clinical presentation) [9] or the Mayo Clinic (based on tumor stage, tumor size, nuclear grade and histological tumor necrosis) [10]. More recently, gene expression signatures have been proposed to add prognostic value to conventional algorithms [11, 12].

The aim of this study was to utilize the vast data resources generated by the Human Protein Atlas project to identify novel biomarkers of clinical relevance for patients with RCC. Cubilin (CUBN) was identified and validated as a marker with the potential to classify RCC patients into low- and high-risk groups, as loss of CUBN expression was significantly and independently associated with less favorable patient outcome. In addition, CUBN expression appears highly specific for RCC compared to other types of cancer, rendering CUBN a possible clinical role in cancer differential diagnostics.

## Methods

### Human Protein Atlas database searches

Global mRNA expression data for 27 normal human tissues [1] was searched for genes specifically expressed in normal kidney and a maximum of six additional tissues. Genes with >5-fold higher fragments per kilobase of transcript per million mapped reads (FPKM) levels in normal human kidney compared to all other tissues and genes with 5-fold higher average FPKM level within a group of 2–7 tissues, including normal human kidney, were investigated further. Corresponding IHC-based expression data within the Human Protein Atlas database (www.proteinatlas.org and unpublished data) was evaluated manually.

Similarly, proteome-wide IHC-based expression data for 83 normal human cell types, corresponding to 44 normal tissues, was searched for proteins expressed in renal tubules or glomeruli and a maximum of nine additional cell types. Retention of protein expression in RCC was evaluated manually. IHC-based expression data for 216 cancer tissues, including up to 12 cases of RCC, were systematically queried for antibodies yielding positive IHC-staining primarily in RCC. Database searches were conducted using varying positive/negative definitions (e.g. negative or weak staining as cut-off) and various levels of specificity (e.g. staining in 50% or 75% of RCC cases and less than 10% or 25% of any other cancer type, respectively).

### Patient cohorts

Initially, a tissue microarray (TMA) containing tumor material from 39 patients with available, corresponding transcriptomics data and protein lysates was used (Additional file 1: Table S1). In addition, three independent TMA cohorts were used. Cohort 1 was a multi-cancer cohort including 940 tumor samples, representing 22 different tumor sites (Additional file 2: Table S2, [13]). Formalin-fixed, paraffin-embedded (FFPE) tumor specimens were identified from the archives of Uppsala University Hospital, Falun Hospital and Lund University Hospital, where all cases were originally diagnosed between 1984 and 2011. A large fraction of samples (502 tumors) represented material from metastatic sites. For RCC, 20 primary tumors and 20 metastases were included. Cohort 2 included 167 primary, 103 venous tumor thrombi and 96 metastatic tumors from 183 RCC patients following radical nephrectomy at the Department of Urology, Edinburgh, between 1983 and 2010 (Additional file 3: Table S3, [14]). Written consent was obtained from study participants from cohort 2. Cohort 3 was assembled from 114 primary ccRCC samples (Additional file 3: Table S3) from patients diagnosed with metastatic RCC between 2006 and 2010 at one of seven Swedish medical centers (Uppsala, Göteborg, Örebro, Västerås, Gävle, Falun, Karlstad). All patients within this cohort had undergone a radical nephrectomy. Written consent was obtained from study participants from cohort 3.

### Tissue microarray construction, immunohistochemistry and annotation

TMAs were constructed as described previously [14, 15]. Two antibodies targeting CUBN were tested (HPA043854 and HPA004133, Atlas Antibodies AB, Stockholm, Sweden). Automated IHC was performed as described previously [15]. IHC staining intensities and fractions of stained tumor cells were manually evaluated and each core annotated by two independent observers. Due to the large number of annotations this task was shared within a group of three observers (TP, NK, GG). Cases with divergent scores were reviewed by a third observer (DD) and consensus reached. Total cellular staining (including cytoplasm and cell membrane) was annotated. Cases were considered positive for CUBN if the fraction of stained cells was greater than 10% and the staining intensity showed at least moderate intensity.

### RNA expression and Western blot analysis

RNA expression analyses were performed as described previously [2]. Western blot analysis was performed according to standard protocols.

### Statistical analysis

For the calculation of sensitivity, specificity and positive predictive value (PPV) standard formulas were applied [16]. Kaplan–Meier survival curves were generated to evaluate the correlation between CUBN expression and patient survival. The log-rank test was used to compare patient survival in groups stratified according to CUBN expression. Cox proportional-hazards regression was applied to estimate hazard ratios in univariate and multivariate models. The *χ* ^2^ test and Fisher’s exact test were used to calculate the significance of associations between CUBN expression and clinicopathological parameters. Calculations were carried out using SPSS Statistics Version 22 (IBM, Armonk, NY).

## Results

### Target identification and antibody validation

**Fig. 1:**
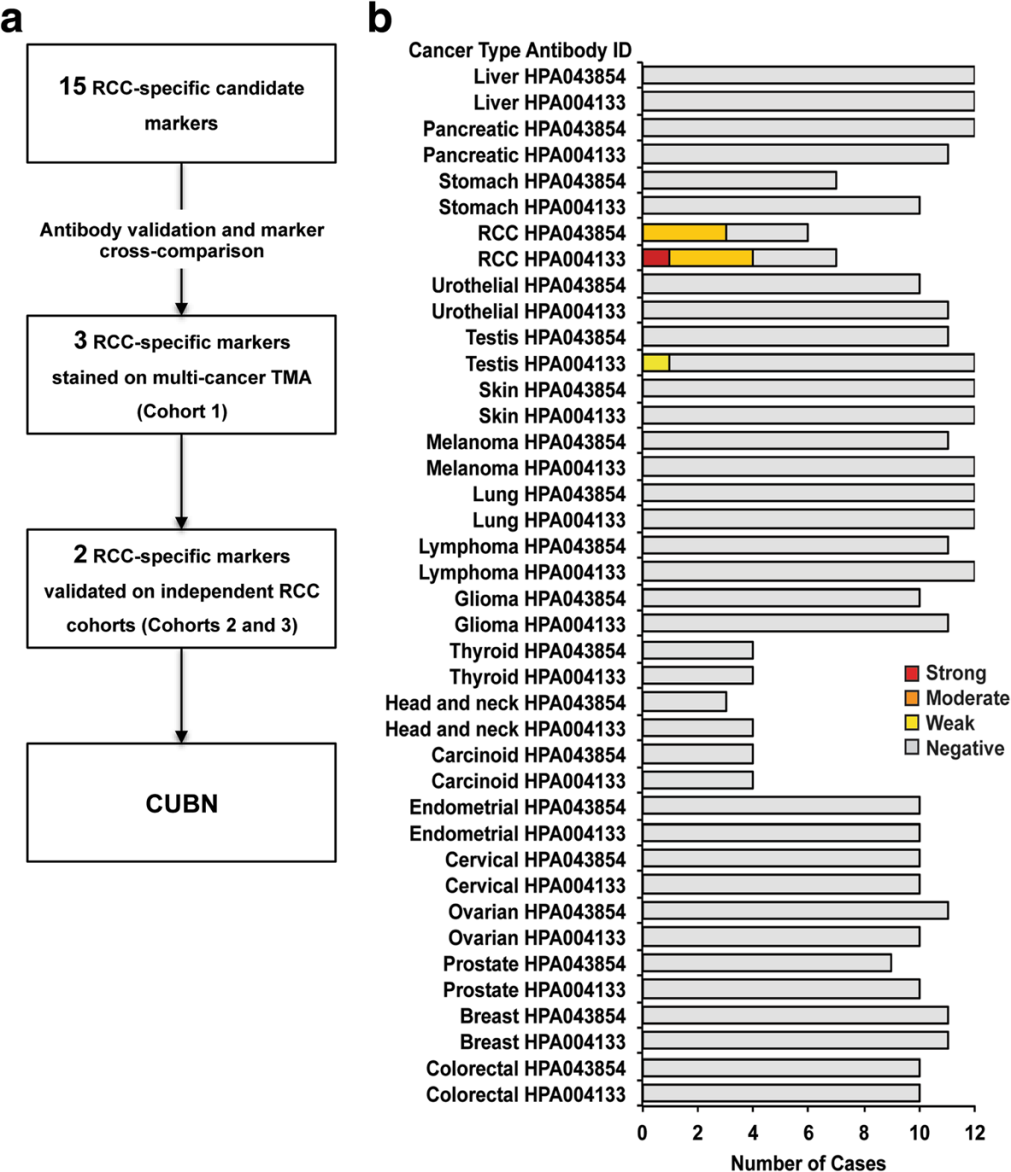
CUBN discovery pipeline and the standard Human Protein Atlas cancer test set. **a** The Human Protein Atlas database (www.proteinatlas.org and unpublished data) was systematically searched for cancer type-specific proteins using automated and manual searches. Staining patterns were reviewed and 15 proteins with RCC-enriched expression chosen for further antibody validation. Following extensive antibody validation and exclusion of antibodies with overlapping staining patterns, three antibodies were selected for validation of RCC-specific staining on multi-cancer TMA cohort 1. Two of these biomarkers were validated further on independent RCC-specific cohorts (cohort 2 and 3) and CUBN identified as highly RCC-specific protein. **b** CUBN staining on routine Human Protein Atlas cancer test set. Two antibodies, HPA004133 and HPA043854, targeting different epitopes on the same protein generated similar staining patterns. Red, orange and yellow coloring indicates cases with strong, moderate and weak staining, respectively. Grey corresponds to CUBN negative cases

The initial focus of this study was to identify kidney-specific proteins whose expression was partly or completely retained in RCC, a prerequisite for an RCC biomarker with prognostic and/or diagnostic value. Following searches within the Human Protein Atlas database, 15 proteins with preferential expression in RCC compared to all other included cancer types were identified (Additional file 4: Table S4). Following systematic antibody validation and immunohistochemical analysis of various TMA cohorts, CUBN was determined as the protein with the highest level of selective expression in RCC (Fig. 1).

**Fig. 2:**
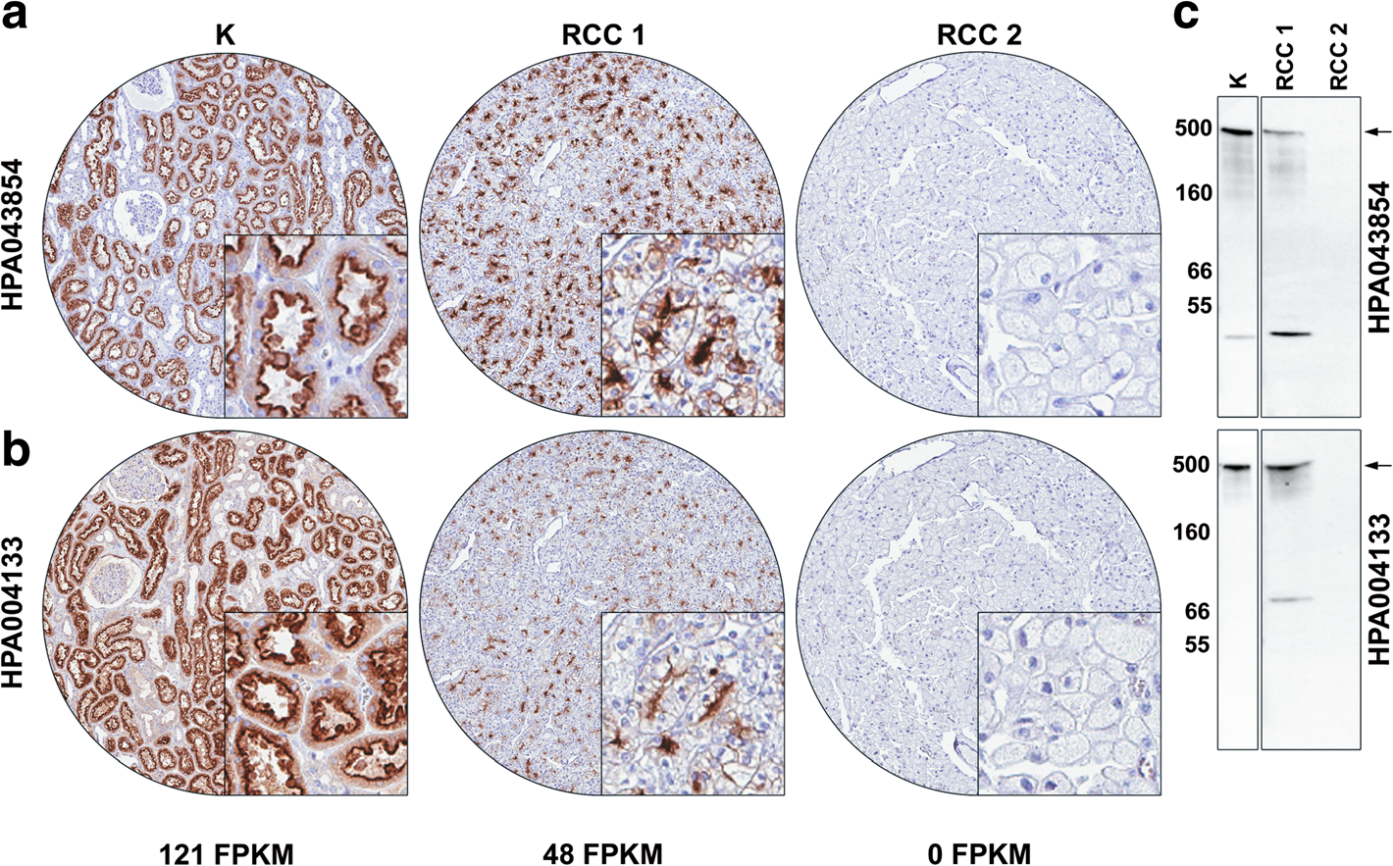
CUBN antibody validation. **a** Two antibodies targeting the CUBN protein at different epitopes (HPA043854 and HPA004133) were tested using immunohistochemistry on a range of normal and malignant tissue. Included in this figure are staining examples from normal human kidney (K) and two renal cell carcinoma cases (RCC1 and RCC2). As chromogen 3,3’-Diaminobenzidine (DAB) was used. **b** RNA-seq expression data from normal human kidney (K) and the renal cell carcinoma cases (RCC1 and RCC2). Expression levels are indicated as fragments per kilobase of exon model per million mapped reads (FPKM). **c** Western blot analysis of CUBN expression in protein extracts from normal human kidney and the renal cell carcinoma cases RCC1 and RCC2 using HPA043854 and HPA004133

Two antibodies targeting CUBN underwent rigorous quality control measures. A comparison of mRNA and IHC-based expression levels in normal human tissues confirmed the specific expression of CUBN in kidney and small intestine (Additional file 5: Figure S1, [17]). Both antibodies specifically stained the proximal tubules of the kidney (Fig. 2a, [18]). Within the test TMA cohort IHC staining intensities correlated well with mRNA expression levels in the same tissues (Fig. 2a and b) and both antibodies produced a Western blot signal at approximately 460 kDa, the molecular weight of CUBN, which was only detected in IHC and RNA positive tissues (Fig. 2c). Additional signals at lower molecular weight were observed for both antibodies in samples with confirmed CUBN expression. These signals were regarded as products of protein degradation. Overall, both antibodies targeting CUBN showed high detection specificity and clone HPA043854 was used for further analyses.

### CUBN as RCC-specific protein

A multi-cancer TMA cohort (cohort 1) was used to substantiate the RCC-specific expression of CUBN. CUBN staining was almost exclusively observed in RCC (Table 1) where 22 out of 39 cases (56%) were annotated as positive. Only one additional case of head and neck cancer (of 20 cases) stained positive for CUBN. This translated to a detection specificity of 100% and PPV of 96% for CUBN in RCC within this cohort.

**Table 1:** CUBN positivity rates on multi-cancer TMA cohort (Cohort 1)

Cancer origin	N (912 total)	CUBN positive N (%^a^)
Prostate	57	0 (0)
Colon	59	0 (0)
Breast	60	0 (0)
Stomach	59	0 (0)
Lung	105	0 (0)
Ovary	60	0 (0)
Endometrium	59	0 (0)
Cervix	59	0 (0)
Hepatocellular	28	0 (0)
Neuroendocrine	30	0 (0)
Sarcoma	60	0 (0)
Urothelial	20	0 (0)
Renal cell carc.	39	22 (56)
- ccRCC	30	18 (60)
- other	9	4 (44)
Lymphoma	20	0 (0)
Melanoma	20	0 (0)
Testis	18	0 (0)
Oesophagus	22	0 (0)
Thyroid	18	0 (0)
Head and Neck	20	1 (5)
Pancreas	51	0 (0)
Cholangiocarc.	41	0 (0)
Gall bladder carc.	7	0 (0)
CUBN specificity^b^		100%
CUBN PPV^b^		96%

*N* number of patients, *ccRCC* clear cell renal cell carcinoma

^a^Percentage of positive cases within tumor type

^b^For RCC compared to all other cases; PPV, positive predictive value

Approximately half of the included RCC samples in cohort 1 were of metastatic origin (20 out of 39 samples) and the expression of CUBN was well maintained in this setting (Additional file 6: Table S5). To further investigate the expression of CUBN during RCC progression, cohort 2 was analyzed. In primary tumors, a similar rate of CUBN positivity (58%) was observed, compared to cohort 1 (Additional file 6: Table S5). However, the number of CUBN positive cases significantly (*P* < 0.001) decreased from venous tumor thrombi with 39% CUBN positivity to metastatic samples with a positivity rate of 29%. Cohort 3 consisted of primary RCC material only with 60% of cases staining positive for CUBN (Additional file 6: Table S5).

### CUBN as marker for good prognosis in ccRCC

**Fig. 3:**
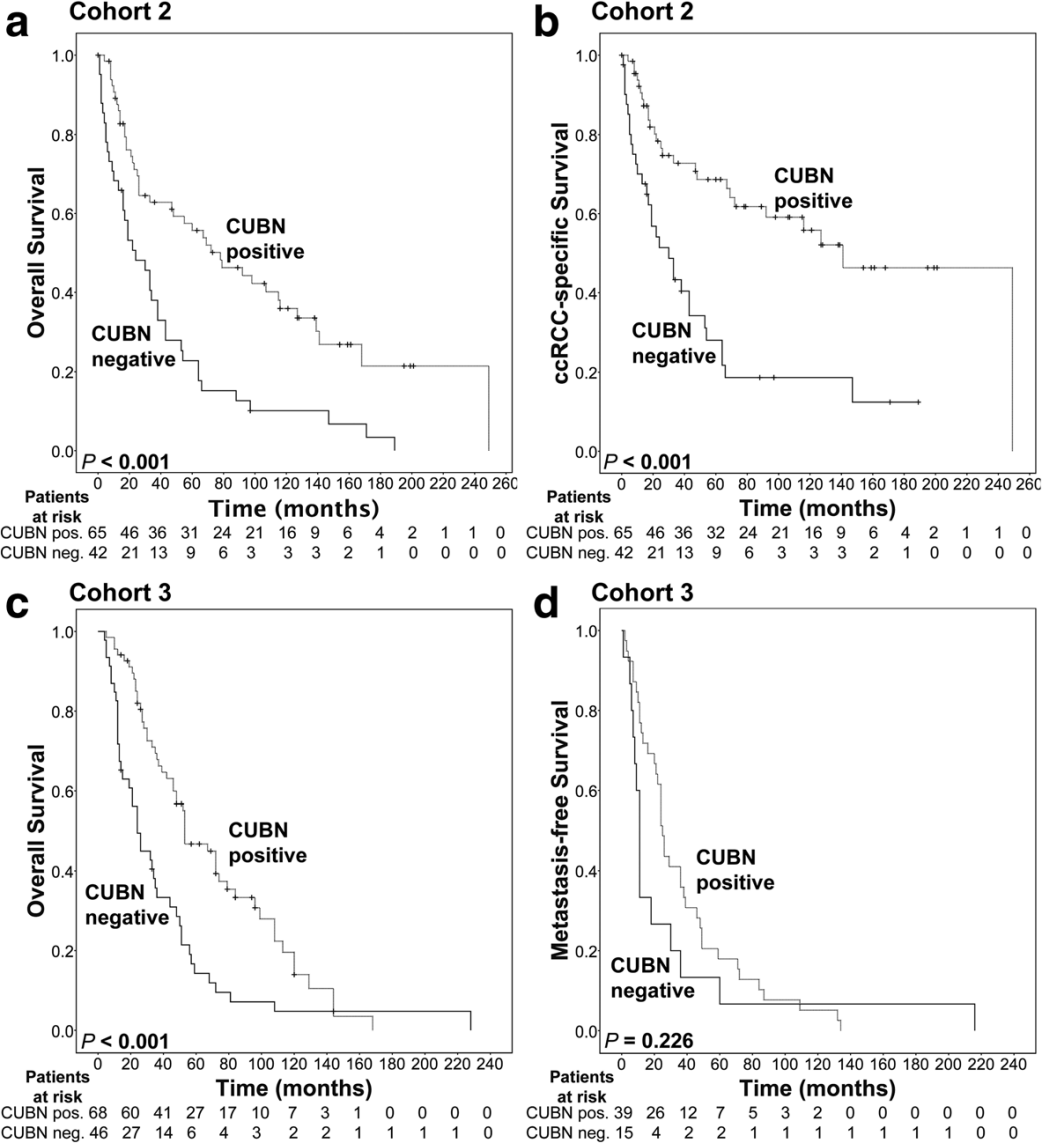
Kaplan-Meier survival analysis of ccRCC patients, stratified according to CUBN expression. **a** Overall survival and **b** ccRCC-specific survival of patients in cohort 2. **c** Overall survival and **d** metastasis-free survival of patients in cohort 3

**Fig. 4:**
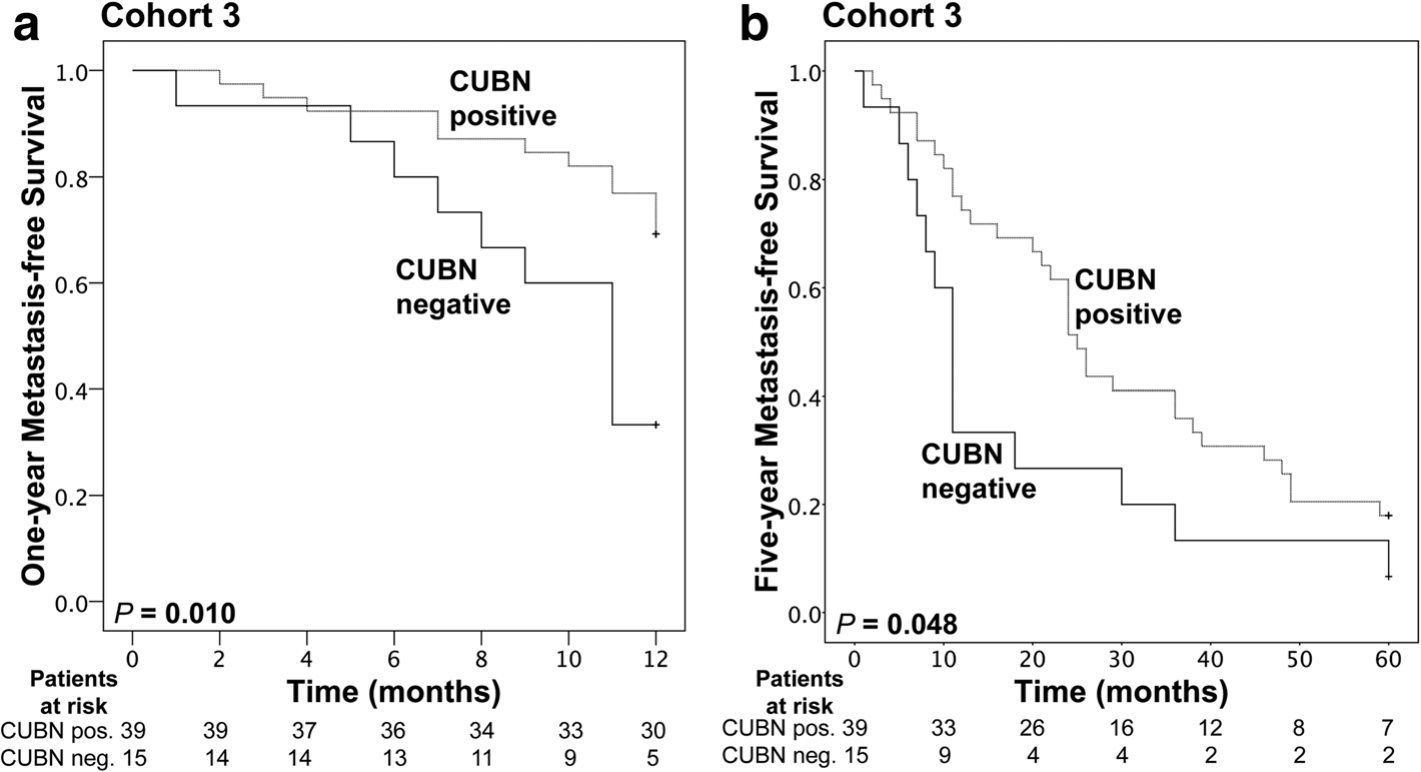
Kaplan-Meier survival analysis of ccRCC patients, stratified according to CUBN expression. **a** One-year metastasis-free survival and **b** five-year metastasis-free survival of patients in cohort 3

Next, we investigated the prognostic relevance of CUBN in RCC. Patient survival information was available for two RCC cohorts (cohorts 2 and 3). Since all cases in cohort 3 and the majority of cases in cohort 2 were ccRCCs, we focused our analyses on this subtype. In cohort 2, stratification of patients according to CUBN positivity showed significant benefit for patients with CUBN positive tumors regarding both, overall survival (*P* < 0.001, Fig. 3a) and ccRCC-specific survival (*P* < 0.001, Fig. 3b). A similar effect was seen in cohort 3, where CUBN positive patient samples were linked to significantly longer overall survival (*P* < 0.001, Fig. 3c). For cohort 3, ccRCC-specific survival information was not available. Instead, the metastasis-free survival of patients initially presenting with localized disease was queried. There was no significant association of metastasis-free survival and CUBN expression overall (*P* = 0.226, Fig. 3d). However, CUBN positive ccRCC patients experienced a significant short-term metastasis-free survival benefit with *P* = 0.01 at 1-year follow-up and *P* = 0.048 at 5-years follow-up (Fig. 4).

### Association of CUBN positivity with clinicopathological parameters and multivariate survival analysis in ccRCC

In cohort 3, positive CUBN staining was significantly associated with localized disease (Table 2, *P* = 0.009). A similar analysis in cohort 2 was not significant (*P* = 0.317). However, this may be due to the small number of patients that presented with distant metastases at diagnosis within this cohort. In cohort 2, the expression of CUBN was related to various other clinicopathological parameters (Table 2). A significant correlation was observed between positive CUBN expression and lower Fuhrman grade (*P* = 0.006) and negative nodal status (*P* = 0.006). No significant association between CUBN expression and T-stage was seen. For cohort 3 similar clinicopathological data were not available.

**Table 2:** Association of CUBN positivity with clinicopathological parameters in ccRCC

Variable	Cohort 2				Cohort 3			
	*N*	CUBN negative *N* (%)	CUBN positive *N* (%)	*P*-value	*N*	CUBN negative *N* (%)	CUBN positive *N* (%)	*P*-value
Spread at diagnosis	131				114			
Local		50 (96)	72 (91)			15 (33)	39 (57)	
Metastatic		2 (4)	7 (9)	0.317^b^		31 (67)	29 (43)	0.009^a^
T-Stage	123							
T1-T2		5 (10)	14 (19)		n.a.			
T3-T4		45 (90)	59 (81)	0.167^a^				
Fuhrman Grade	95							
1–2		8 (23)	31 (52)		n.a.			
3–4		27 (77)	29 (48)	0.006^a^				
Nodal Status	131							
Negative		39 (75)	73 (92)		n.a.			
Positive		13 (25)	6 (8)	0.006^a^				

*N* number of patients

^a^χ^2^ test

^b^Fisher’s exact test; n.a., not available

Univariate Cox regression analysis confirmed the relevance of CUBN as good prognostic marker for overall survival (Table 3, HR 0.411, 95% CI 0.263–0.641, *P* < 0.001), and ccRCC-specific survival (Additional file 7: Table S6, HR 0.334, 95% CI 0.199–0.569, *P* < 0.001). The association remained significant in multivariate analysis following adjustment for T-stage, Fuhrman grade and nodal status for both, overall survival (Table 3, HR 0.382, 95% CI 0.203–0.719, *P* = 0.003) and ccRCC-specific survival (Additional file 7: Table S6, HR 0.297, 95% CI 0.142–0.620, *P* = 0.001).

**Table 3:** Cox regression analysis of overall survival (Cohort 2)

Prognostic factor	Univariate			Multivariate^a^		
	HR	(95% CI)	*P*-value	HR	(95% CI)	*P*-value
CUBN (pos. vs. neg., ref)	0.411	0.263–0.641	<0.001	0.382	0.203–0.719	0.003
T-Stage (T3-T4 vs. T1-T2, ref)	1.897	1.002–3.593	0.049	1.689	0.746–3.825	0.209
Fuhrman Grade (3–4 vs. 1–2, ref)	1.822	1.059–3.136	0.030	1.217	0.665–2.226	0.524
Nodal Status (pos. vs. neg., ref)	4.208	2.397–7.386	<0.001	4.041	1.840–8.874	0.001

*HR* hazard ratio, *CI* confidence interval

^a^Adjusted for all other variables; pos., positive; neg., negative; ref, referent group

## Discussion

We utilized the Human Protein Atlas resources to identify in an unbiased fashion, novel targets to improve and supplement currently used tools for the prognostication and differential diagnosis of RCC. Following state-of-the-art validation of antibodies targeting CUBN [19], we analyzed the expression of CUBN in normal human tissues, a large variety of cancers and two RCC-specific cohorts. We found that loss of CUBN expression in ccRCC patients was significantly associated with poor prognosis. Importantly, this observation was independent of T-stage, Fuhrman grade and nodal status, implying added clinical value of routine CUBN testing. In addition, we found the expression of CUBN to be highly specific to RCC, suggesting a potential use of CUBN in clinical cancer differential diagnostics as a complement to other diagnostic antibodies in cases where RCC needs to be confirmed.

CUBN is an endocytic receptor that is specifically expressed on epithelial cells in the proximal tubules of the kidney and in glandular cells of the small intestine [20]. In the kidney, CUBN mediates the reabsorption of filtered proteins such as albumin and transferrin [18], whereas in the small intestine, CUBN is primarily involved in the uptake of intrinsic factor-vitamin B_12_ complex [21]. Even though the role of CUBN in normal kidney and small intestine has been well characterized and CUBN has been used as a marker for renal cell differentiation [22], the role of CUBN during RCC development and progression is largely unknown.

Although IHC is not quantitative, results from validated antibodies provide protein expression data at cellular resolution and can readily be translated to a clinical setting. The applied TMA methodology also appears well suited to simulate small tissue biopsies, which are exceedingly relevant in the clinical practice. The specificity and sensitivity of IHC staining for CUBN in cohorts of tumor tissue has provided an example of a novel diagnostic biomarker for RCC. Although extended studies regarding the expression pattern in additional tumors of relevance for differential diagnostics, e.g. adrenal gland tumors and other forms of clear cell cancer, are required to establish the usefulness of CUBN staining in clinical routine, the presented results indicate that this marker could be used for difficult cases where a diagnosis of RCC needs to be confirmed.

There is an unmet need for better tools for risk stratification of ccRCC patients. Several prognostic algorithms based on clinicopathological parameters have been proposed. For example, algorithms developed at Memorial Sloan-Kettering Cancer Center [9] or the Mayo Clinic [10] are used for the prediction of recurrence in patients with localized ccRCC. More recently, molecular phenotyping of RCC has shown promise in adding prognostic value to standard clinicopathological parameters. With ClearCode34, a 34-gene expression signature for the prognostic stratification of localized ccRCC patients was introduced and a combination of molecular and clinical parameters shown to provide better risk prediction than clinical variables alone [11]. Unlike mRNA-based assays, the immunohistochemical detection of CUBN can easily be implemented in routine pathology laboratories. An application of CUBN as marker for early disease spread and the added value of CUBN as a prognostic marker over clinical stage, grade and nodal status are promising and additional validation is highly desirable.

Functional studies to understand the mechanism linking the expression of a protein involved in re-absorption of proteins in proximal tubules and aggressiveness of RCC are needed. Previous studies showing that TGF beta reduces CUBN expression [23] and contributes to RCC aggressiveness [24] could provide one starting point to explore the biological background for the correlation between CUBN expression in RCC and prognosis. Extended functional studies regarding malignancy grade and also larger studies on well-defined cohorts with high quality clinical data from RCC patients will be needed to further explore the role of CUBN in RCC and to establish the clinical utility of this promising RCC biomarker.

## Conclusions

In a quest to identify novel biomarkers for RCC, we have applied a systematic search strategy to exploit the extensive data resources of the Human Protein Atlas (www.proteinatlas.org). We identified CUBN as a marker for risk stratification of patients with RCC. Lack of CUBN expression was significantly associated with early disease progression and poor patient outcome, independent of T-stage, Fuhrman grade and nodal status. Owing to a highly RCC-specific expression profile, CUBN expression also has a potential role in clinical cancer differential diagnostics.

## Additional files

Additional file 1: Table S1. Test TMA cohort composition. (DOC 34 kb)
Additional file 2: Table S2. Cohort 1 sample characteristics. (DOC 63 kb)
Additional file 3: Table S3. Available clinicopathological parameters of primary tumors in cohort 2 and cohort 3. (DOC 31 kb)
Additional file 4: Table S4. RCC-specific candidate biomarkers. (DOC 33 kb)
Additional file 5: Figure S1. Comparison of CUBN mRNA and IHC-derived protein expression in normal tissue. mRNA and protein expression levels were indicated as a percentage of the maximum. IHC-derived expression values were assigned numerical values; three for strong, two for moderate and one for weak staining. Staining intensities were averaged over the number of available tissue microarray cores (three cores per tissue type). (TIF 1128 kb)
Additional file 6: Table S5. CUBN positivity rates according to tumor site. (DOC 30 kb)
Additional file 7: Table S6. Cox regression analysis of ccRCC-specific survival (Cohort 2). (DOC 30 kb)

## Abbreviations

ccRCC: Clear cell renal cell carcinoma
CUBN: Cubilin
DAB: 3,3’-Diaminobenzidine
FFPE: Formalin-fixed, paraffin-embedded, PPV, Positive predictive value
FPKM: Fragments per kilobase of transcript per million mapped reads
HR: Hazard ratio
IHC: Immunohistochemistry
PAX2: Paired box protein 2
PAX8: Paired box protein 8
RCC: Renal cell carcinoma
TGF beta: Transforming growth factor beta
TMA: Tissue microarray
