# Supplementary material for: A systematic search strategy identifies cubilin as independent prognostic marker for renal cell carcinoma
Source: BMC Cancer. 2017 Jan 4;17:9. doi: 10.1186/s12885-016-3030-6 (PMC5215231; doi:10.1186/s12885-016-3030-6)
Supplement: Additional file 1: Table S1. — Test TMA cohort composition. (DOC 34 kb) [file 12885_2016_3030_MOESM1_ESM.doc]

**Table S1** Test TMA cohort composition

| **Tumor Site** | **N** |
| --- | --- |
| Prostate | 4 |
| Colorectal | 4 |
| Breast | 4 |
| Lung | 1 |
| Ovary | 4 |
| Endometrial | 4 |
| Hepatocellular | 2 |
| Urothelial | 2 |
| **Renal cell carcinoma** | **3** |
| Melanoma | 1 |
| Testis | 4 |
| Pancreas | 4 |
| Cholangiocarcinoma | 2 |
| **Total** | **39** |

N, number of included cases
