# Supplementary material for: A systematic search strategy identifies cubilin as independent prognostic marker for renal cell carcinoma
Source: BMC Cancer. 2017 Jan 4;17:9. doi: 10.1186/s12885-016-3030-6 (PMC5215231; doi:10.1186/s12885-016-3030-6)
Supplement: Additional file 2: Table S2. — Cohort 1 sample characteristics. (DOC 63 kb) [file 12885_2016_3030_MOESM2_ESM.doc]

**Table S2** Cohort 1 sample characteristics

| **Tumor Site** | **Sex**  **F M** | | **Metastasis Samples** | **Primary Tumor Samples** | **Recurrence Samples** | **Total** |
| --- | --- | --- | --- | --- | --- | --- |
| Prostate | - | 60 | 26 | 31 | 3 | 60 |
| Colorectal | 23 | 37 | 50 | 10 | 0 | 60 |
| Breast | 59 | 1 | 50 | 10 | 0 | 60 |
| Stomach | 21 | 39 | 47 | 13 | 0 | 60 |
| Lung |  |  |  |  |  |  |
| - Adenocarcinoma | 28 | 36 | 32 | 32 | 0 | 64 |
| - Squamous cell | 12 | 24 | 14 | 22 | 0 | 36 |
| - Lung NOS | 6 | 3 | 4 | 5 | 0 | 9 |
| Ovary | 60 | - | 50 | 10 | 0 | 60 |
| Endometrial | 60 | - | 18 | 40 | 2 | 60 |
| Cervical | 60 | - | 22 | 36 | 2 | 60 |
| Hepatocellular | 9 | 21 | 0 | 29 | 1 | 30 |
| Neuroendocrine | 18 | 12 | 30 | 0 | 0 | 30 |
| Sarcoma | 35 | 25 | 32 | 26 | 2 | 60 |
| Urothelial | 9 | 11 | 20 | 0 | 0 | 20 |
| **Renal cell carcinoma** |  |  |  |  |  |  |
| **- ccRCC** | **8** | **23** | **14** | **17** | **0** | **31** |
| **- other** | **3** | **6** | **6** | **3** | **0** | **9** |
| Lymphoma | 6 | 14 | 0 | 20 | 0 | 20 |
| Melanoma | 8 | 12 | 20 | 0 | 0 | 20 |
| Testis | - | 19 | 8 | 11 | 0 | 19 |
| Esophagus | 6 | 16 | 6 | 16 | 0 | 22 |
| Thyroid | 13 | 5 | 10 | 8 | 0 | 18 |
| Head and neck | 8 | 12 | 13 | 6 | 1 | 20 |
| Pancreas | 27 | 33 | 19 | 41 | 0 | 60 |
| Cholangiocarcinoma | 24 | 20 | 8 | 36 | 0 | 44 |
| Gall bladder | 5 | 3 | 3 | 5 | 0 | 8 |
| **Total** | **508** | **432** | **502** | **427** | **11** | **940** |

F, female; M, male; ccRCC, clear cell renal cell carcinoma; Table adapted from Gremel et al., Histopathology. 2014 Jan;64(2):293-305
