# Supplementary material for: A systematic search strategy identifies cubilin as independent prognostic marker for renal cell carcinoma
Source: BMC Cancer. 2017 Jan 4;17:9. doi: 10.1186/s12885-016-3030-6 (PMC5215231; doi:10.1186/s12885-016-3030-6)
Supplement: Additional file 3: Table S3. — Available clinicopathological parameters of primary tumors in cohort 2 and cohort 3. (DOC 31 kb) [file 12885_2016_3030_MOESM3_ESM.doc]

**Table S3** Available clinicopathological parameters of primary tumors in cohort 2 and cohort 3

| **Variable** | **Cohort 2**  **N (%)** | **Cohort 3**  **N (%)** |
| --- | --- | --- |
| **Median age (range)** | 64.5 years (38-94) | 63 years (33-77) |
| **Median follow-up (range)** | 33 months (0-249) | 36.5 months (4-228) |
| **Gender**  male  female | 96 (60)  65 (40) | 81 (71)  33 (29) |
| **T-stage**  T1  T2  T3  T4 | 10 (7)  13 (9)  119 (81)  4 (3) | n.a. |
| **Fuhrman grade**  1  2  3  4 | 3 (3)  41 (38)  46 (43)  17 (16) | n.a. |
| **Nodal status**  negative  positive | 137 (83)  28 (17) | n.a. |
| **Histology**  ccRCC  other | 135 (91)  14 (9) | 114 (100) |
| **Spread at diagnosis**  local  metastatic | 153 (93)  12 (7) | 54 (47)  60 (53) |

n.a., not available; ccRCC, clear cell renal cell carcinoma
