# Supplementary material for: A systematic search strategy identifies cubilin as independent prognostic marker for renal cell carcinoma
Source: BMC Cancer. 2017 Jan 4;17:9. doi: 10.1186/s12885-016-3030-6 (PMC5215231; doi:10.1186/s12885-016-3030-6)
Supplement: Additional file 4: Table S4. — RCC-specific candidate biomarkers. (DOC 33 kb) [file 12885_2016_3030_MOESM4_ESM.doc]

**Table S4** RCC-specific candidate biomarkers

| **Target** | **Cohorts Analysed** |
| --- | --- |
| **CUBN** (cubilin) | Test TMA, TMA Cohorts 1-3 |
| **ADSSL1** (adenylosuccinate synthase Like 1) | Test TMA, TMA Cohorts 1-3 |
| **PKLR** (pyruvate kinase, liver and RBC) | Test TMA, TMA Cohort 3 |
| **c4orf49** (chromosome 4 open reading frame 49) | Test TMA |
| **GLYAT** (glycine-N-acyltransferase) | Test TMA  Test TMA |
| **CDH16** (cadherin 16) | Test TMA |
| **PIRT** (phosphoinositide-interacting regulator of transient receptor potential) | Test TMA |
| **MLEC** (malectin) | Test TMA |
| **ENPEP** (glutamyl aminopeptidase (aminopeptidase A)) | Test TMA |
| **DMGDH** (dimethylglycine dehydrogenase) | Test TMA |
| **METTL7B** (methyltransferase like 7B) | Test TMA |
| **SCGN** (secretagogin) | Test TMA |
| **MFRP** (membrane frizzeled related protein) | Test TMA |
| **ITGB3** (integrin, beta 3) | Test TMA |
| **WDR6** (WD repeat domain 6) | Test TMA |
