# Supplementary figures and images for: A systematic search strategy identifies cubilin as independent prognostic marker for renal cell carcinoma
Source: BMC Cancer. 2017 Jan 4;17:9. doi: 10.1186/s12885-016-3030-6 (PMC5215231; doi:10.1186/s12885-016-3030-6)

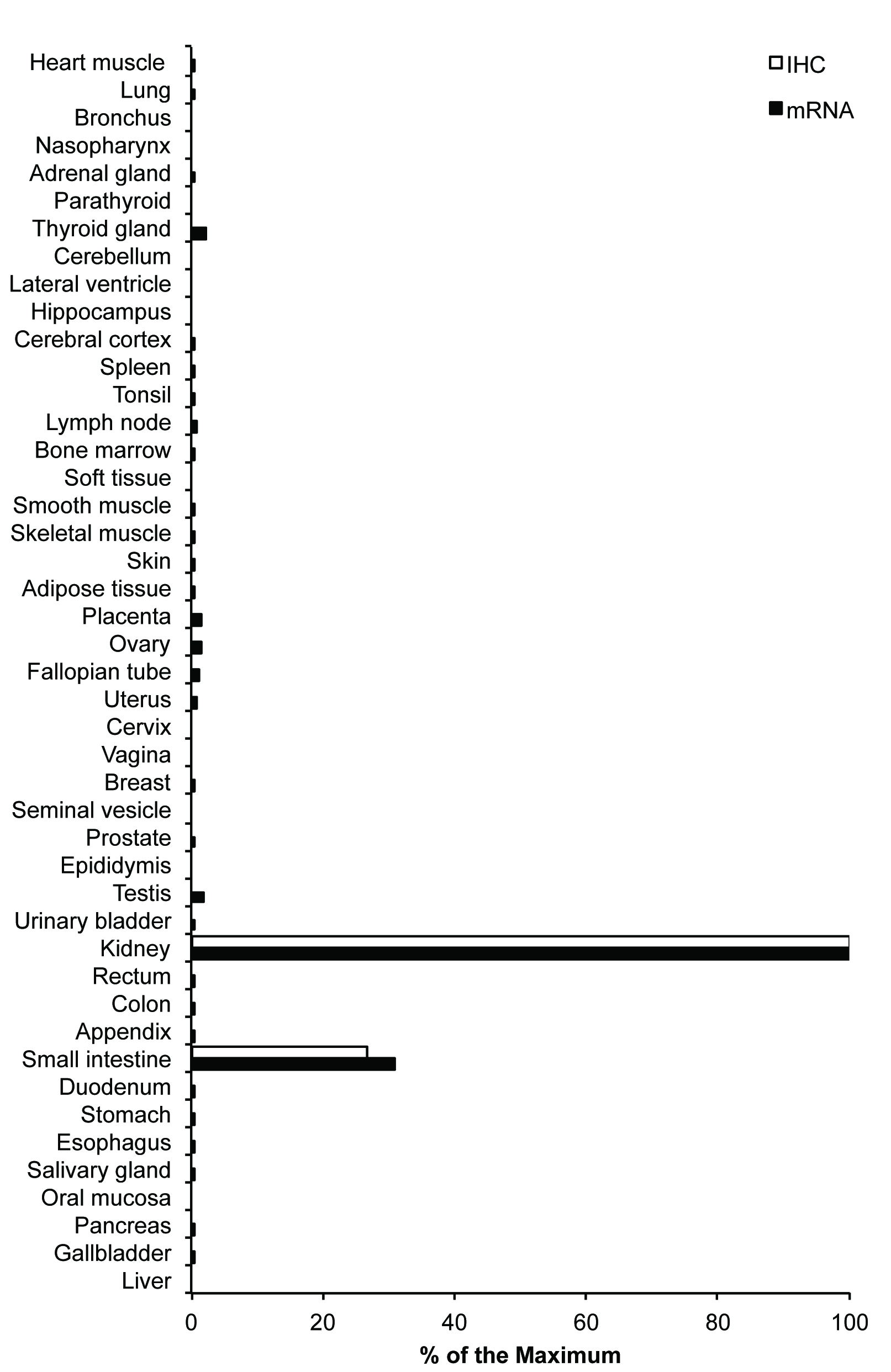

Supplement: Additional file 5: Figure S1. — Comparison of CUBN mRNA and IHC-derived protein expression in normal tissue. mRNA and protein expression levels were indicated as a percentage of the maximum. IHC-derived expression values were assigned numerical values; three for strong, two for moderate and one for weak staining. Staining intensities were averaged over the number of available tissue microarray cores (three cores per tissue type). (TIF 1128 kb) [file 12885_2016_3030_MOESM5_ESM.tif]
