# Supplementary material for: A systematic search strategy identifies cubilin as independent prognostic marker for renal cell carcinoma
Source: BMC Cancer. 2017 Jan 4;17:9. doi: 10.1186/s12885-016-3030-6 (PMC5215231; doi:10.1186/s12885-016-3030-6)
Supplement: Additional file 6: Table S5. — CUBN positivity rates according to tumor site. (DOC 30 kb) [file 12885_2016_3030_MOESM6_ESM.doc]

**Table S5** CUBN positivity rates according to tumour site

| **Cohort** | **Site** | **N** | **CUBN Positive**  **N (%^a^)** |
| --- | --- | --- | --- |
| Cohort 1 | Primary | 19 | 10 (52) |
|  | Metastatic | 20 | 12 (60) |
| Cohort 2 | Primary | 160 | 92 (58) |
|  | Venous tumor thrombus | 92 | 36 (39) |
|  | Metastatic | 62 | 18 (29) |
| Cohort 3 | Primary | 114 | 68 (60) |

N, number of patients; ^a^percentage of positive cases within tumor site
