# Supplementary material for: A systematic search strategy identifies cubilin as independent prognostic marker for renal cell carcinoma
Source: BMC Cancer. 2017 Jan 4;17:9. doi: 10.1186/s12885-016-3030-6 (PMC5215231; doi:10.1186/s12885-016-3030-6)
Supplement: Additional file 7: Table S6. — Cox regression analysis of ccRCC-specific survival (Cohort 2). (DOC 30 kb) [file 12885_2016_3030_MOESM7_ESM.doc]

**Table S6** Cox regression analysis of ccRCC-specific survival (Cohort 2)

| **Prognostic Factor** | **Univariate** | | |  | **Multivariate^a^** | | |
| --- | --- | --- | --- | --- | --- | --- | --- |
|  | HR | (95% CI) | *P*-value |  | HR | (95% CI) | *P*-value |
| CUBN (pos. vs. neg., ref) | 0.334 | 0.196-0.569 | **<0.001** |  | 0.297 | 0.142-0.620 | **0.001** |
| T-Stage (T3-T4 vs. T1-T2, ref) | 1.576 | 0.772-3.218 | 0.212 |  | 1.199 | 0.483-2.972 | 0.696 |
| Fuhrman Grade (3-4 vs. 1-2, ref) | 2.326 | 1.203-4.497 | **0.012** |  | 1.509 | 0.720-3.165 | 0.276 |
| Nodal Status (pos. vs. neg., ref) | 4.714 | 2.562-8.675 | **<0.001** |  | 4.081 | 1.748-9.527 | **0.001** |

^a^Adjusted for all variables except Nodal Status; pos., positive; neg., negative; dist., distant; loc., local; ref, referent group; HR, hazard ratio; CI, confidence interval
